# SARS-CoV-2 nomenclature: viruses, variants and vaccines need a standardized naming system

**DOI:** 10.2217/fvl-2021-0198

**Published:** 2021-11-04

**Authors:** Tariq Khan, Syed Muhammad Jamal

**Affiliations:** ^1^Department of Biotechnology, University of Malakand, Chakdara, Dir Lower, KP, Pakistan

**Keywords:** coronavirus, COVID-19, nomenclature, SARS-CoV-2, vaccines, variant of concern

The issue of naming the coronavirus had arisen about twenty years back and the need for a standard nomenclature system was asserted after the emergence of SARS in 2002–2003 [1]. However, the issue resurfaced in early 2020 when a novel coronavirus (SARS-CoV-2), deadlier than the previous, brought the world to a halt. Over the years, no solid standard naming system has been developed and implemented.

Unlike the binomial nomenclature, a system for naming other organisms (plants, animals and bacteria etc.), there is a lack of a standardized naming convention for viruses. For researchers who are constantly discovering new viruses, the lack of a naming convention has been frustrating. Due to this reason, viruses are classified based on characteristics such as structure of their capsid (e.g., SARS-CoV-2), presence, or absence of an envelope (i.e., enveloped or nonenveloped virus), type of nucleic acid in their genome (i.e., DNA or RNA virus), their strategy of translating nucleic acids coding to proteins (in case of RNA viruses, e.g., positive sense or negative sense), their host range, clinical signs (e.g., SARS CoV), organs affected (e.g., foot-and-mouth disease virus), pathogenicity and location of first detection (e.g., Congo–Crimean hemorrhagic fever virus). Sequence comparisons and ascertaining phylogenetic relationship have now become the pillars of viral taxonomy.

Based on the above features, a hierarchical relationship is developed that groups together viruses with similar properties. For example, viruses of the order Nidovirales are all enveloped, positive-strand RNA viruses. The family Coronaviridae (order Nidovirales) comprises of a single large 26–32 Kb genome and large (∼20 nm) petal-shaped surface projections which form an image resembling the solar corona [2].

Coronaviruses belong to the family Coronaviridae. There are four genera within this family, in other words, *Alpha*-, *Beta*-, *Delta*- and *Gammacoronavirus*. The *Alpha*- and *Betacoronaviruses* primarily infect mammals, whereas *Delta*- and *Gammacoronaviruses* primarily infect swine and birds, respectively.

SARS-CoV-2, the causative agent of COVID-19, is a species within the genus *Betacoronavirus*. Other species within this genus are SARS-CoV, the causative agent of SARS pandemic in 2002–2004 and the causative agent of the Middle East respiratory syndrome. After its initial discovery in late 2019, SARS-CoV-2 spread throughout the world. Four different variants of this virus have so far been described, in other words, B.1.1.7, B.1.351, P.1 and B.1.617.2 that are also referred to as the UK, South Africa, Brazil and Indian variants, respectively [3].

However, virus nomenclature based on the location of its first detection or nationality causes problems such as diplomatic crisis, stigmatization and even racial profiling, among other things [4]. Because of this, the WHO and other health regulatory bodies have argued that the coronavirus should not be given a name that is associated with people or a specific location. In general, the longer it takes to name a virus, the more long-lasting a fictitious name becomes. This appears to be a minor issue in comparison to the potentially lethal consequences of the virus's spread. However, the significance of proper naming cannot be undermined.

The International Committee on Taxonomy of Viruses (ICTV) has now approved and ratified a binomial genus-species naming system for virus species. This approval came in March 2021 after the meeting of ICTV Executive Committee in October 2020 [5]. The ICTV has suggested that all previously established species names be converted to the new format. However, as of now, no proper implementation measures have been stated. Researchers, the media, and government officials continue to call the virus names that ignore the new ICTV rules. This is due in part to the complexity of the binomial nomenclature. While bringing binomial nomenclature to viral taxonomy is a positive step, these names are very difficult for the public. For everyday use, short and catchy names like Measles are highly recommended. The WHO should urge governments, the media, and other public entities to continue to refer to the virus such as ‘the coronavirus’, while avoiding any other derogatory terms. The scientific community should, however, adopt the new nomenclature as soon as possible.

## Naming variants: a new confusion

When it is suggested that political correctness is important while naming deadly viruses, it is because some entities are happy to call them by names that stigmatize those places and people. A recent example is that of the new variant ‘B.1.617.2’ dubbed ‘Indian variant’ as reported in a New Scientist Magazine article [6]. Similarly, the previous variants B.1.1.7, B.1.351 and P.1 are referred to as the UK, South Africa and Brazil variants, respectively [3]. Such nomenclature results in racism and xenophobia. For example, calling the variant with names such as ‘Indian variant’ has sparked a wave of prejudice against Indians. To avoid these issues, the four coronavirus variants that the WHO considers as variants of concern, have now been assigned the Greek letters Alpha, Beta, Gamma and Delta, in order of their detection [7]. Other proposals, such as naming the variants after Greek gods, fruits, plants or acronyms for the phrase ‘variant of concern’ (VOC1, VOC2), were discussed but rejected by the panelists for a variety of reasons. According to the WHO, the new names (Alpha, Beta, Gamma and Delta) are intended to be simple and avoid prejudice, and they will not replace existing scientific names (such as B.1.1.7, B.1.351 and P.1 assigned by Pangolin [8], which convey important scientific information.

This is a good first step. However, there is an issue with assigning variants the Greek letters. In attempting to avoid one problem, the WHO has ended up causing another. As stated earlier, SARS-CoV-2 belongs to the genus *Betacoronaviruses*. The names of the variants of SARS-CoV-2, in other words, Alpha, Beta, Gamma and Delta have created another confusion with the names of the genera (Supplementary Figure 1). While deciding on these names, the confusion with assigning variants names that already correspond to the coronavirus genera should have been considered. As the goal was to achieve ease of use and simplicity, naming the variants in a much simpler and straightforward manner, such as variant A (Var-A), variant B (Var-B), variant C (Var-C), and so on, could have been a better option.

## Vaccines, just like viruses, need a standardized & easy naming system

Just like naming SARS-CoV-2 and its variants, there has also been some confusion about the names of the vaccines. Currently, 21 COVID vaccines have been approved for use in at least one country (Supplementary Table 1). The WHO has given emergency use authorization to six of these vaccines [9].

Name of the manufacturer, the method of development, and the country of development are all used interchangeably to identify these vaccines. This has created confusion. For example, people confuse the terms “SinoVac” and “CoronaVac” vaccines, when CoronaVac is a vaccine manufactured by SinoVac Biotech Ltd., a Chinese biopharmaceutical company. Furthermore, Sinopharm, a biopharmaceutical company, has developed BBIBP-CorV and WIBP-CorV, two traditionally inactivated vaccines. Both the vaccines have been approved for emergency use. BBIBP-CorV has been dubbed more effective than WIBP-CorV [10]. It has also been approved by the WHO. However, both vaccines are referred to as Sinopharm, with no distinction made between them. Similarly, the Ad26.COV2.S vaccine was developed by Janssen Pharmaceuticals, a subsidiary of Johnson & Johnson, a multinational corporation. This vaccine is also named, interchangeably, as the Janssen or Johnson & Johnson vaccine [11,12], which ultimately causes confusion among the general public.

Vaccine refusal is as old as the vaccines themselves. It takes an inordinate amount of effort, resources, and time to persuade people to get vaccinated [13]. Just as health authorities, governments, and the media are responsible for promoting vaccination, they are also responsible for naming the vaccines in a consistent manner to avoid confusion.

In conclusion, an effective system of nomenclature should be in place to standardize names of viruses, variants, and vaccines as quickly as possible. The ICTV should act swiftly to bring these names forward. To avoid prejudice, misconceptions and stigmatization related to such nomenclatures, governments, public entities, and the media should act responsibly in accordance with the conventions.

## Future perspective

Because of the world’s deadliest outbreak caused by SARS-CoV-2, virus nomenclature is expected to take a serious turn. Furthermore, it is expected that more than 100,000 complete SARS-CoV-2 genome sequences will be generated in near future. It is also expected that more variants will emerge soon, necessitating a very clearly laid out nomenclature system to avoid confusion. In addition to variants, new viruses, more lethal than the current one, may emerge and quickly spread around the world. Failure to prepare in every aspect of virus biology may allow the next epidemic to be far more devastating than the current one. Naming viruses and variants according to their geographical location of first detection or nationality in which they were first observed, and given the fact that the developed world is rapidly recovering, there is a risk of stigmatization growing in relation to variants emerging in developing countries. It is expected that significant efforts will be made to establish an easy-to-use standard nomenclature system for viruses.
