# Supplementary material for: SARS-CoV-2 nomenclature: viruses, variants and vaccines need a standardized naming system
Source: Future Virol. 2021 Nov 4:10.2217/fvl-2021-0198. doi: 10.2217/fvl-2021-0198 (PMC8577721; doi:10.2217/fvl-2021-0198)
Supplement: Supplementary file 1 [file Supplementary_information.docx]

Supplementary Material


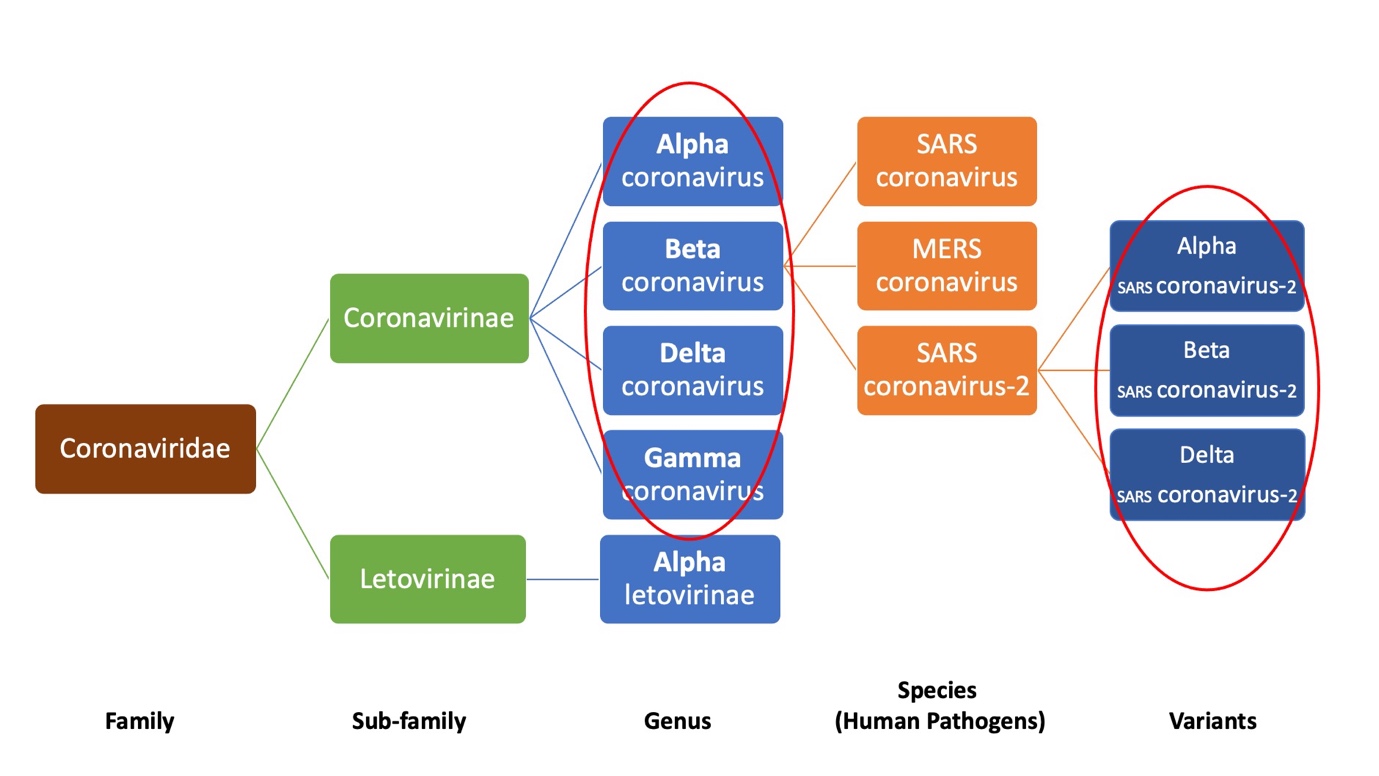


Figure S1: Classification of coronaviruses. Illustration of how the new names of the variants are causing confusion with names assigned to genera in the family Coronaviridae

Table S1: Details of the authorized/approved vaccines against COVID-19

| **S.No** | **Name** | **Vaccine type** | **Primary developer** | **Country or Origin** | **Authorization type** |
| --- | --- | --- | --- | --- | --- |
|  | Comirnaty (BNT162b2) | mRNA-based vaccine | Pfizer, BioNTech; Fosun Pharma | Multinational | WHO |
|  | Moderna COVID‑19 Vaccine (mRNA-1273) | mRNA-based vaccine | Moderna, BARDA, NIAID | US | WHO |
|  | AstraZeneca (AZD1222); aka Vaxzevria and Covishield | Adenovirus vaccine | BARDA, OWS | UK | WHO |
|  | Sputnik V | Recombinant adenovirus vaccine (rAd26 and rAd5) | Gamaleya Research Institute, Acellena Contract Drug Research and Development | Russia | Various countries* |
|  | Sputnik Light | Recombinant adenovirus vaccine (rAd26) | Gamaleya Research Institute, Acellena Contract Drug Research and Development | Russia | Various countries* |
|  | Janssen (JNJ-78436735; Ad26.COV2.S) | Non-replicating viral vector | Janssen Vaccines (Johnson & Johnson) | The Netherlands, US | WHO |
|  | CoronaVac | Inactivated vaccine (formalin with alum adjuvant) | Sinovac | China | WHO |
|  | BBIBP-CorV | Inactivated vaccine | Beijing Institute of Biological Products; China National Pharmaceutical Group (Sinopharm) | China | WHO |
|  | EpiVacCorona | Peptide vaccine | Federal Budgetary Research Institution State Research Center of Virology and Biotechnology | Russia | Various countries* |
|  | Convidicea (PakVac, Ad5-nCoV) | Recombinant vaccine (adenovirus type 5 vector) | CanSino Biologics | China | Various countries* |
|  | Covaxin (BBV152) | Inactivated vaccine | Bharat Biotech, ICMR; Ocugen; ViroVax | India | Various countries* |
|  | WIBP-CorV | Inactivated vaccine | Wuhan Institute of Biological Products; China National Pharmaceutical Group (Sinopharm) | China | China |
|  | CoviVac | Inactivated vaccine | Chumakov Federal Scientific Center for Research and Development of Immune and Biological Products | Russia | Russia |
|  | ZF2001 (ZIFIVAX) | Recombinant vaccine | Anhui Zhifei Longcom Biopharmaceutical, Institute of Microbiology of the Chinese Academy of Sciences | China, Uzbekistan | China, Uzbekistan |
|  | QazVac (QazCovid-in) | Inactivated vaccine | Research Institute for Biological Safety Problems | Kazakhstan | Kazakhstan |
|  | Unnamed vaccine candidate | Inactivated vaccine | Minhai Biotechnology Co.; Kangtai Biological Products Co. Ltd. | China | China |
|  | COVIran Barekat | Inactivated vaccine | Shifa Pharmed Industrial Group | Iran | Iran |
|  | Unnamed vaccine candidate | Inactivated vaccine | Chinese Academy of Medical Sciences, Institute of Medical Biology | China | China |
|  | Abdala (CIGB 66) | Protein subunit vaccine | Center for Genetic Engineering and Biotechnology | Cuba | Cuba |
|  | Soberana 02 | Conjugate vaccine | Finlay Institute of Vaccines; Pasteur Institute | Cuba, Iran | Cuba, Iran |
|  | MVC-COV1901 | Protein subunit vaccine | Medigen Vaccine Biologics Corp.; Dynavax | Taiwan | Taiwan |

*The information about different countries could be found on this website which was used for the data in this table (<https://www.raps.org/news-and-articles/news-articles/2020/3/covid-19-vaccine-tracker>).
